# Triplication of the Sigmoid: A Rare Incidental Finding in Association With an Anorectal Malformation During Colostomy Closure

**DOI:** 10.7759/cureus.54000

**Published:** 2024-02-11

**Authors:** Ahmed Zubar Zain, Sara Zuheir Fadil, Hussein Naji

**Affiliations:** 1 Pediatric Surgery, College of Medicine, Al-Nahrain University, Baghdad, IRQ; 2 Pediatric Surgery, Central Child Teaching Hospital, Baghdad, IRQ; 3 Pediatric Surgery, Mohammed Bin Rashid University of Medicine and Health Sciences, Dubai, ARE; 4 Pediatric Surgery, Mediclinic Parkview Hospital, Dubai, ARE

**Keywords:** diagnostic challenges, rare anomalies, colostomy closure, anorectal malformation, triplication of sigmoid

## Abstract

This case report presents a rare occurrence of triplication of the sigmoid, an unusual congenital anomaly, in a nine-month-old male with a known history of anorectal malformation. The patient, previously diagnosed with anal atresia and a rectourethral (prostatic) fistula, was admitted for the closure of his divided sigmoidostomy as the final step in correcting his anorectal malformation. Unexpectedly, during the release of the distal stoma, the presence of three distinct bowel lumens was discovered. To discern the native bowel, catheters were introduced into each lumen before proceeding with the excision of the triplicated sigmoid and subsequent stoma closure. This case underscores the complexity of diagnosing and managing unusual GI anomalies in the context of anorectal malformations, emphasizing the challenges encountered during surgical interventions.

## Introduction

Alimentary tract duplications are congenital anomalies that can occur anywhere along the GI tract, ranging from the mouth to the anus. While large bowel duplications are relatively uncommon, they still represent around 10% of all alimentary tract duplications [1]. The first recorded case of intestinal duplication was documented by Calder in 1733. However, it was not until 200 years later, in 1937, that William E. Ladd published a significant report on the topic. Ladd identified three key characteristics to define these congenital lesions: a well-developed smooth muscle layer, an epithelial lining resembling GI mucosa, and an intimate anatomical connection with a portion of the GI tract [2]. Among the various types of enteric duplication, triplication of the small intestine stands out as an exceedingly rare variant. Although instances of gastric and colonic triplications have been sparsely documented in the literature, they remain infrequent occurrences [3,4]. Numerous theories have been proposed to elucidate the underlying mechanisms responsible for the formation of GI tract duplications. These theories include aberrant luminal recanalization, embryonic diverticula, a split notochord, and partial twinning. However, to date, no single theory has offered a comprehensive explanation for all types of duplications. The complexity of this phenomenon suggests that multiple factors may be involved in the development of these congenital lesions [5-7]. In this article, we present a unique case report of sigmoid triplication in association with anorectal malformation discovered during the closure of a colostomy. This intriguing case sheds light on the rarity and complexity of GI tract triplications, adding to the growing body of knowledge concerning these congenital anomalies.

## Case presentation

**Figure 1:**
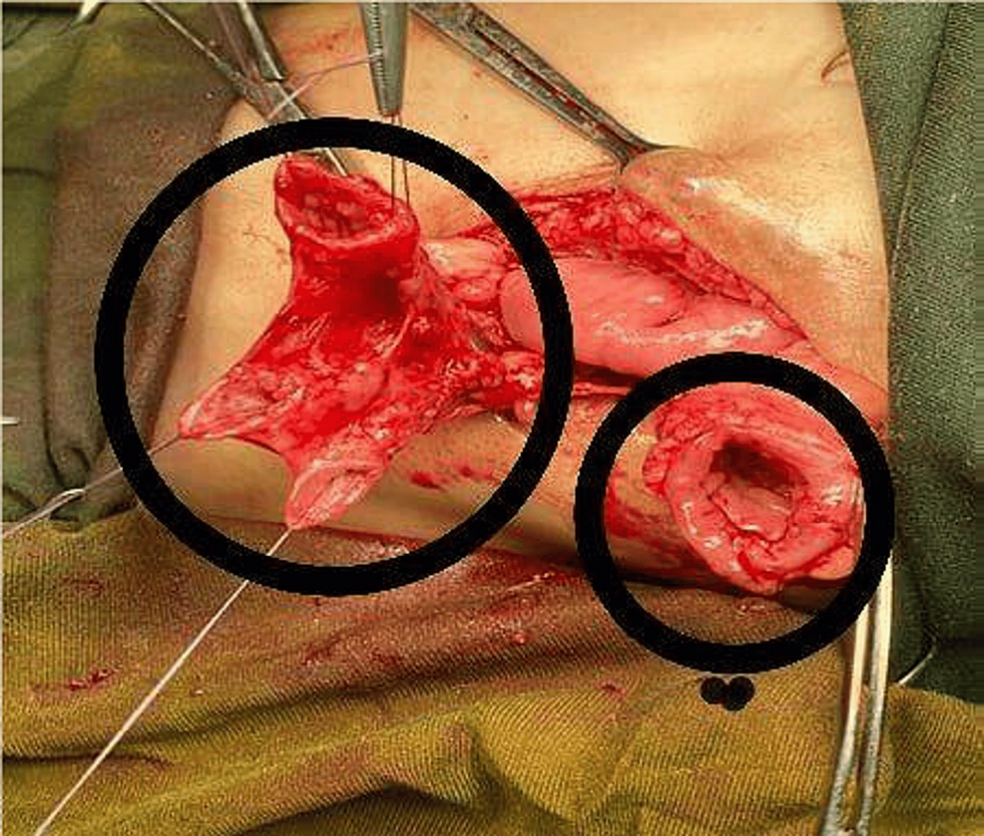
Proximal sigmoid loop in the small circle; the triplicated sigmoid (distal loop) in the large circle.

A nine-month-old male infant, previously diagnosed with anorectal malformation and a rectourethral fistula (prostatic fistula), was admitted to our pediatric surgical ward for elective closure of his divided sigmoidostomy as the third stage of surgical correction for his anorectal malformation. Upon complete release of the distal stoma during the surgical procedure, an unexpected discovery was made: there were three distinct bowel lumens, revealing a tubular triplication of the distal sigmoid (Figure 1).

**Figure 2:**
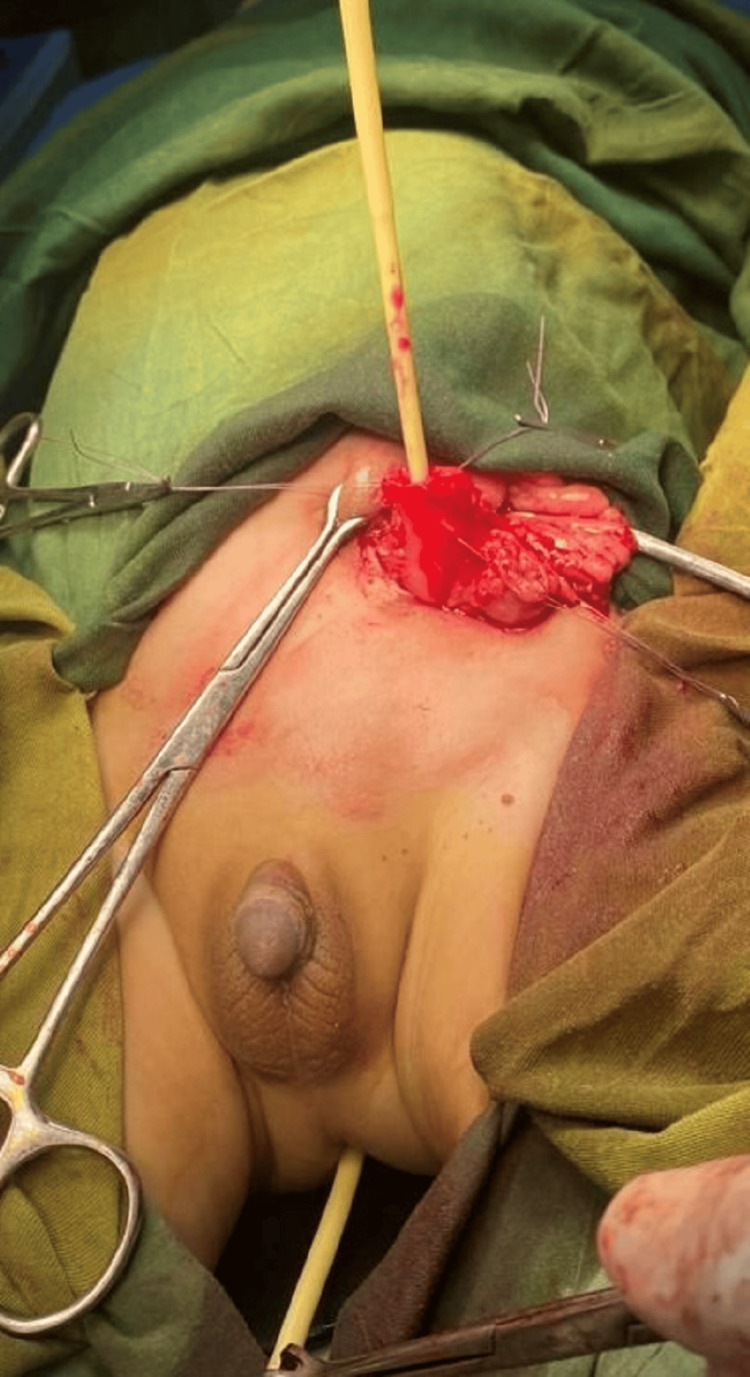
Foley catheter inserted through the native distal sigmoid loop.

**Figure 3:**
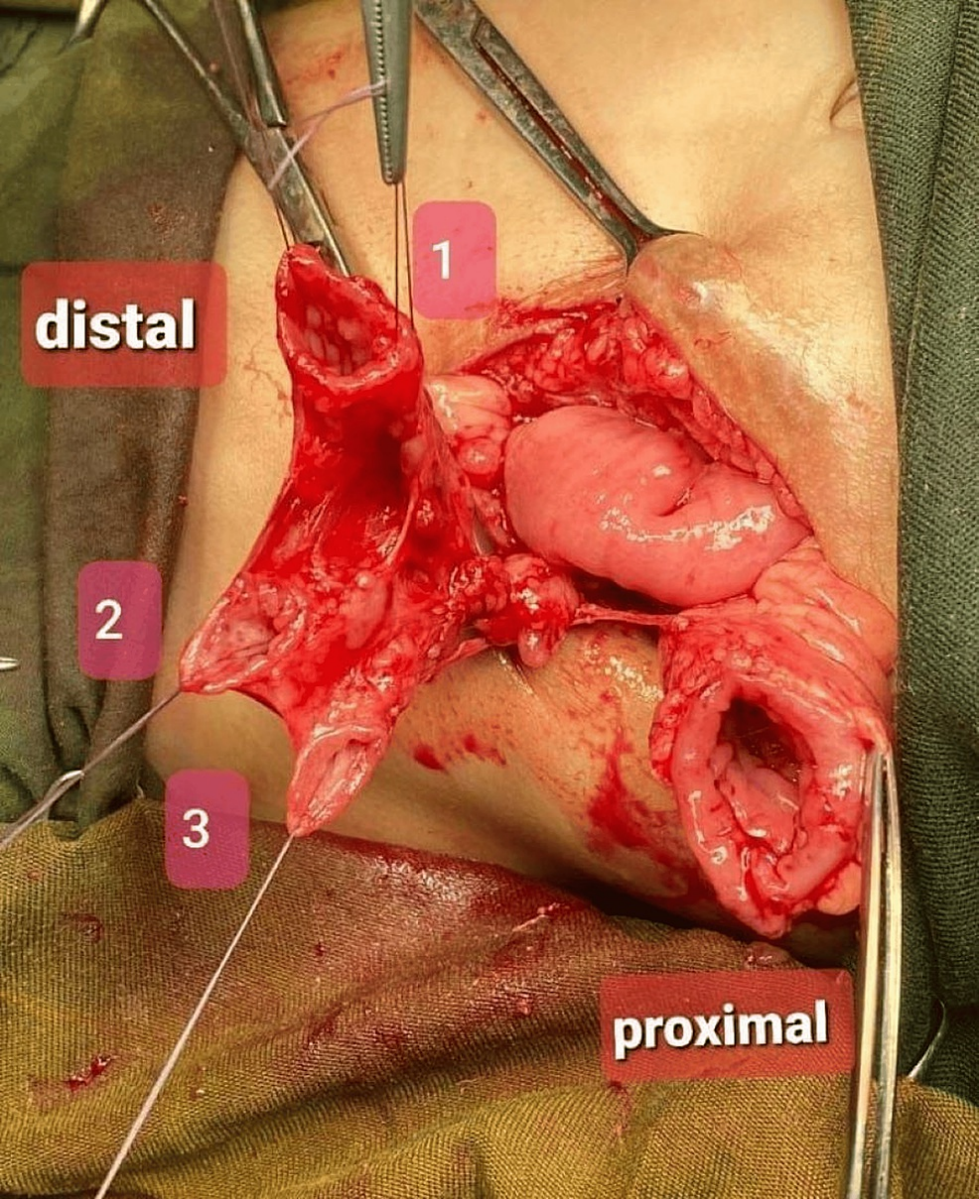
Triplicated tubular sigmoid (1 is the native loop, 2 is the duplicate, and 3 is the triplicate).

In order to proceed with the proper closure of the sigmoidostomy and preserve the native intestine (sigmoid), catheters were carefully inserted into each lumen to identify the native bowel segment (Figures 2-3). Subsequently, the duplicated intestinal portion was excised, and the native intestine was preserved. A one-layer end-to-end anastomosis was meticulously performed to close the sigmoid.

Histopathological examination of the excised bowel confirmed the presence of two separate segments of the sigmoid, providing definitive evidence of the triplication anomaly.

Following the surgical intervention, the child's recovery was uneventful. He was discharged on the fourth postoperative day after successfully initiating oral feeding without experiencing abdominal distension or any complications. During the six-month follow-up period, the patient remained in excellent health and showed no signs of adverse effects or recurrence. This extraordinary case underscores the importance of recognizing and managing rare gastrointestinal anomalies in conjunction with anorectal malformation, contributing to improved patient outcomes, and raising awareness within the medical community about these unusual clinical scenarios.

## Discussion

Congenital malformations of the digestive system, including various atresias, malformations of fixation, anorectal malformations, and intestinal duplications, exhibit an overall prevalence of 1.3 per 1000 [7]. Triplications of the alimentary tract represent an exceptionally rare anomaly, with only a few reported cases in the literature, typically involving the esophagus, stomach, or colon [8-10].

Among GI triplications, triplication of the colon is an exceedingly rare occurrence, with only three cases reported in the medical literature [4,11,12]. The patient discussed in this report, a known case of anorectal malformation (anal atresia with a rectourethral fistula), was admitted for elective closure of the sigmoidostomy. Incidentally, during the surgical procedure, triplication of the sigmoid was unexpectedly discovered, similar to the diagnostic path observed in the previous three reported cases of colonic triplications.

The diagnosis of intestinal duplications and triplications can present challenges. Contrast studies may reveal indirect signs, such as compression of the intestinal wall or contrast-filled duplications when communication is present. USG and CT scans are helpful adjuncts, potentially demonstrating cystic or elongated structures with thick walls [13]. In the current case, preceding the corrective surgery for the anorectal malformation, a contrast study was administered through the distal opening of the colostomy. Regrettably, this study did not disclose the existence of the triplication. Given the rarity of this condition, there was a low level of suspicion at that time, leading to the decision not to pursue further imaging studies.

In the present case, the surgical excision of the triplicated sigmoid, coupled with the closure of the colostomy, was successfully executed without encountering any complications. Surgical excision is commonly recommended once the diagnosis is made, aiming to prevent complications and perform the procedure in the optimal state of the patient. However, there is a difference of opinion, as some experts suggest that only symptomatic duplications should undergo surgical treatment. The standard surgical procedure entails excision of the duplication [14].

Colorectal duplications are typically benign lesions, which is why surgical resection should be non-radical, aiming for complete resection of the duplication along with the involved portion of the colon. Treatment, usually in the form of surgical resection, is reserved for symptomatic cases. The decision for surgical intervention depends on the acute setting, the presence of symptoms, the type of duplication, and any associated anomalies [15].

## Conclusions

Triplication of the sigmoid is an exceptionally rare congenital anomaly, especially when it occurs in conjunction with an anorectal malformation. This unique condition poses significant challenges in terms of preoperative diagnosis and investigation, presenting a complex puzzle for pediatric surgeons. The rarity of such cases complicates their recognition and proper management during the preoperative phase. Continued reporting and study of these cases are essential to expand our knowledge and contribute to improved care for affected individuals in the future.
